# Supplementary material for: The Prevalence and Comorbidity of Tic Disorders and Obsessive-Compulsive Disorder in Chinese School Students Aged 6–16: A National Survey
Source: Brain Sci. 2022 May 16;12(5):650. doi: 10.3390/brainsci12050650 (PMC9139904; doi:10.3390/brainsci12050650)
Supplement: Supplementary file 1 [file brainsci-12-00650-s001.zip › brainsci-1700909-SI.pdf]

The Study Protocol of National-Scale Psychiatric Epidemiological Point Prevalence Survey for School-Going Children and Adolescents in China

Main Structure: Four parts were included in this section. The first part was about the method of determine the target sample size [1]; the second part was about the sample procedures; the third part was about the assessment procedure; and the last part was the statistical analysis.

Sample Size

To determine the target sample size, we considered the prevalence of Tourette's disorder, which was reported to have one of the lowest prevalences among children and adolescents aged 6–17 years in the United States: ~0.30%. [2]. With relative error (ε) of 15% and a confidence coefficient of 95% (Z), the sample estimation would be 56,742 (statistic power = 1). Assuming the proportion of those lost to follow-up was 20%, the required sample size would be 70,927. Formula of sample size calculation.

$$n = \frac{Z^2_{1-\frac{\alpha}{2}}(1-p)}{p\varepsilon^2}$$

Sample Procedures

In China, the age range of school children and adolescents is from 6 to 17 years (primary school: 6–12 years, junior middle school: 12–16 years, and senior middle school: 16–19 years). For logistical reasons, a decision was made to exclude adolescents, 17 years of age, who were in their final year of senior middle school, given the demands of the College Entrance Examination. The target population of this study was set between 6 and 16 years of age. In 2015, the enrollment rate of primary school, junior middle school, and senior middle school in China was 99.88% (net enrollment rate), 104% (gross enrollment rate), and 87% (gross enrollment rate), respectively (Ministry of Education of People's Republic of China, 2016), according to a multistage cluster stratified random sampling strategy. There are 34 provincial administrative regions in China, including 23 provinces, 5 autonomous regions, 4 municipalities (Beijing, Shanghai, Tianjin, and Chongqing), and 2 special administrative regions (Hong Kong and Macau). Five provincial administrative regions were selected as the sampling locations (Beijing, Hunan, Jiangsu, Liaoning, and Sichuan). These locations were selected by comprehensively considering geographical partition, economic development, and rural/urban factors. The geographical partition was defined in an official document (National Bureau of Statistics of China, 2011), which divided the country into northeastern, eastern, middle, and western regions. Liaoning, Jiangsu, Hunan, and Sichuan were selected as representatives of these regions, respectively. For the economic development factor, we aimed to investigate the differences between high-income (gross national

income per capita more than 12,736 US dollars (Fantom & Serajuddin, 2016)) megacities (population over 10 million (State Council of the People's Republic of China, 2014)) (developed areas) and other areas (developing areas). In 2015, among 658 cities (including county-level, prefectural-level, sub-provincial, and municipalities) throughout the country, 43 cities had a gross domestic product per capita (roughly equals to gross national income per capita) over USD 12,736, and there were 13 cities that had a population larger than 10 million. Six cities were found in the intersection of these two sets. They are Shenzhen, Guangzhou, Suzhou, Tianjin, Beijing, and Shanghai. We did not sample in Suzhou city, and we excluded the weights of Suzhou in the statistics process related to Jiangsu Province and developing areas. Beijing was chosen as the representative of the developed areas. The urban/rural category for each sampling location was defined by the Provisions of the Statistical Division of Urban and Rural Areas (National Bureau of Statistics, 2006). There are 13 prefectural level administrative divisions in Beijing with a population of 2.55 million, aged 6–16 years; 14 divisions in Hunan with a population of 8.54 million, aged 6–16 years; 13 divisions in Jiangsu with a population of 10.27 million, aged 6–16 years; 14 divisions in Liaoning with a population of 5.69 million, aged 6–16 years; and 20 divisions in Sichuan with a population of 10.45 million, aged 6–16 years.

Provinces	Beijing	Liaoning	Liaoning	Hunan	Sichuan	Total
Primary/Middle schools	15/13	16/18	15/17	18/17	21/19	85/74
Number of classes	345	361	337	354	367	1764
Selected individuals	10,549	12,116	13,531	17,071	20,725	73,992

Figure S1: the first stage of sample procedure.

Assessment procedure

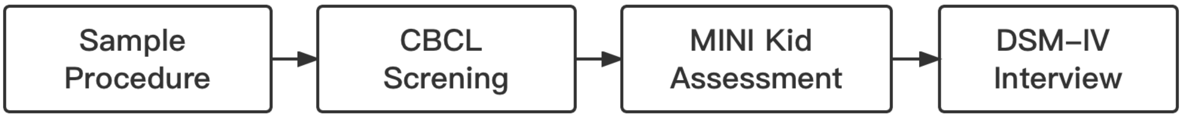

Figure S2: The Assessment procedure. (Note: CBCL: the Child Behavior Checklist; MINI-Kid: Mini-International Neuropsychiatric Interview for Children and Adolescents; DSM-IV: the Diagnostic and Statistical Manual of Mental Disorders, Fourth Edition.)

A two-phase epidemiological survey was conducted, including questionnaire screening and the interview stage. In the first stage, a total of 73,992 participants aged 6-16 years were enrolled by cluster sampling and screened by CBCL in the first stage. In the second stage, a detailed psychiatric assessment was performed for individuals with

the high-risk score of CBCL. Each family was interviewed twice by clinicians. The first interview was conducted with the Mini International Neuropsychiatric Interview for Children and Adolescents (MINI-Kid), and the second interview was a DSM-IV based interview. The rating results of the first interview were provided to the clinicians conducting the second interview as references. A final diagnosis was made after the DSM--IV based interview in Stage 2.

Statistical analysis

Point-weighted prevalence for 26 mental disorders was then estimated. We adjusted prevalence estimates with the product of sampling weights and poststratification weights. Standard error values and 95% confidential intervals were generated with Taylor series linearization (Korn et al., 1998). The overall sampling weight for each participant in Stage 1 was the product of sampling weights of their provincial region, prefectural division, county/district, school, and class. In Stage 2, the weights of randomly picked participants with negative CBCL screening results were multiplied with the reciprocal of their sampling probabilities. Poststratification weight was calculated by utilizing demographic characteristics of participants: (a) age group (6–11 years and 12–16 years), (b) sex, and (c) location of residence (urban vs. rural). Nonresponse adjustment was included in the poststratification process. Individuals who refused to participate or did not have their primary caregivers finish the CBCL were treated as nonrespondents.

We used the reciprocal of response rate in the corresponding demographic sub-group of each participant as their nonresponse weights. Sociodemographic characteristic comparisons between all participants and the nonrespondents were performed by using exact binomial tests. The tests were conducted to determine if the nonrespondent quit students were nonrandom of the total sample with regard to their sociodemographic characteristics (Table S1). The population information for poststratification was taken from the 2010 national census. Rao–Scott adjusted chi-square ($\chi2$) tests were employed to compare the prevalence estimates of diagnosed mental disorders between demographic groups. The Rao–Scott adjusted $\chi2$ test is a modified $\chi2$ test method for survey data, which improves the performance in the goodness-of-fit problem and can help to avoid misleading results generated by clustering in the survey design (Rao & Scott, 1987). The significance of the results was then adjusted by the false discovery rate (Benjamini & Hochberg, 1995). A logistic regression for any diagnosis was used to examine the independent variables of age group, sex, area development level, and interactions among them. Data analysis was performed with the R language 3.4.0.

Table S1. The descriptive statistic for the 8 syndromes of the CBCL for the whole sample.

Syndromes	Mean	SD	Skewness	Kurtosis
A/D(AD)	2.08	2.839	2.123	6.039
W/D(WD)	1.94	2.142	1.436	2.354
SC	1.97	2.499	1.806	4.250
SP	2.30	2.483	1.504	2.965
TP	1.90	2.550	2.446	9.512
AP	2.68	2.551	1.063	0.933
RBB	1.71	2.086	2.241	8.970
AB	4.36	4.324	1.460	2.770
Total problems	22.54	20.497	1.664	5.190

Note: AD: Anxious/Depressed; W/D: Withdrawn/Depressed; SC: Somatic Complaints; SP: Social Problems; TP: Thought Problems; AP: Attention Problems; RBB: Rule-Breaking Behavior; AB: Aggressive Behavior.

Table S2. The number of different types of comorbidities for OCD.

Types of Comorbidities	Total Number	Boys vs. Girls	Children vs. Adolescents
Comorbidities with Tic Disorders	216	79 vs. 137	186 vs. 30
Comorbidities with Affective Disorders	83	25 vs. 58	15 vs. 68
Comorbidities with ADHD	42	23 vs. 19	20 vs. 22

References

1. Li F, Cui Y, Li Y, Guo L, Ke X, Liu J, Luo X, Zheng Y, Leckman JF: Prevalence of mental disorders in school children and adolescents in China: diagnostic data from detailed clinical assessments of 17,524 individuals. *Journal of Child Psychology and Psychiatry* **2022**, *63*, 34–46.
2. Control CfD, Prevention: Prevalence of diagnosed Tourette syndrome in persons aged 6-17 years-United States**, 2007**. *MMWR Morbidity and mortality weekly report* 2009, *58*, 581–585.
